# Identity Reconstruction as a Coping Mechanism in Addiction Recovery: A Pilot Stratified Randomized Controlled Trial of Narrative Therapy Group Intervention

**DOI:** 10.3390/ejihpe16050068

**Published:** 2026-05-14

**Authors:** Peipei Wang, Yanan Li, Xu Cheng, Hong Xie, Huanxian Huang, Jun Yang, Yangwei Chen, Alex Pak Ki Kwok, Jiacheng Chen

**Affiliations:** 1 Mental Health Education Center, Zhuhai Campus, Jinan University, Zhuhai 519070, China; peipeiwang@jnu.edu.cn; 2 Physical Education Department, Zhuhai Campus, Jinan University, Zhuhai 519070, China; liyanan@jnu.edu.cn; 3 Department of Psychology, College of Arts and Sciences, Beijing Normal University, Zhuhai 519087, China; 4 Zhuhai Compulsory Isolation Drug Rehabilitation Center, Zhuhai 519080, China; 5 Data Science and Policy Studies Programme, School of Governance and Policy Science, Faculty of Social Science, The Chinese University of Hong Kong, Hong Kong 999077, China; 6 College of Education for the Future, Beijing Normal University at Zhuhai, Zhuhai 519087, China

**Keywords:** narrative therapy, identity reconstruction, substance use disorder, group intervention, psychological relapse risk, evidence-based practice, coping mechanisms

## Abstract

Relapse in substance use disorders remains a persistent clinical challenge. Identity reconstruction, the psychological transition from an internalized “addict” identity to a recovery-oriented self, may be a core coping mechanism for abstinence maintenance. This exploratory pilot stratified randomized controlled trial examined the feasibility and preliminary effects of a Narrative Therapy (NT) group intervention, delivered within an evidence-based practice framework. Forty-five male residents of a closed rehabilitation facility were randomly assigned (*n* = 15 each) to an 8-week NT intervention, standard psychoeducation (TAU), or waitlist control (WLC). The Self-Identity Scale and an abbreviated four-item Stimulant Relapse Risk Scale served as outcomes. The Self-Concept Clarity Scale assessed a candidate process variable. A 3 (group) × 3 (time) repeated-measure ANOVA showed significant group × time interactions for self-identity (*F* = 64.215, *p* < 0.001, and *η^2^p* = 0.754) and relapse-risk indicators (*F* = 62.131, *p* < 0.001, and *η^2^p* = 0.747). For self-identity, only the NT group showed significant within-group gains. For relapse-risk indicators, NT scores were significantly lower than both control groups at post-test and follow-up (all pairwise *p* ≤ 0.008), with these reductions maintained at two-month follow-up. Within-group self-concept clarity gains emerged for NT, though between-group differences were nonsignificant. Because the study was conducted in a custodial setting, the relapse-risk findings reflect psychological vulnerability rather than observed behavior. Larger adequately powered trials are needed.

## 1. Introduction

Addiction is more than a disorder of the brain. It is a crisis of identity (Gligorov & Cowan, 2025). Neurobiological models have advanced our understanding of substance dependence, yet they capture only part of the clinical picture. The persistent challenge of relapse, often described as a “revolving door” phenomenon (Di Giovanni et al., 2020), suggests that biological detoxification alone is insufficient. Individuals who complete detoxification programs frequently return to substance use, not because withdrawal management has failed, but because they remain psychologically tethered to an identity defined by addiction (Best et al., 2016). This psychosocial dimension of recovery warrants systematic investigation.

Recent policy developments in China illustrate a broader global shift in how addiction treatment is conceptualized. The nation’s approach to substance use has undergone the following paradigm transformation: from punitive isolation models emphasizing compulsory detention toward human-centric frameworks prioritizing community rehabilitation and social reintegration (Song et al., 2026). This transition reflects an emerging consensus that recovery is not merely physiological but fundamentally social. The individual must be restored not only as a biological organism free from chemical dependence but as a “social being” capable of meaningful participation in family, work, and community life. Yet despite these policy advances, high relapse rates persist, signaling that current interventions may be missing a critical target.

A growing body of evidence identifies this target as identity. Individuals with substance use histories are frequently defined, both by society and by themselves, through a singular, stigmatized label: the “addict.” This label, analyzed in Goffman (1963)’s foundational work on stigma, eclipses all other social roles. The person ceases to be recognized as a parent, friend, colleague, or neighbor; they become, in their own eyes and in the eyes of others, reducible to their addiction. Such identity foreclosure generates the following profound psychological consequences: internalized shame, social withdrawal, and a pervasive sense of defectiveness and hopelessness. These are not peripheral features of the addiction experience. They constitute a self-reinforcing cycle that erodes motivation for change and elevates relapse risk (McIntosh & McKeganey, 2000).

Theoretical and empirical scholarship increasingly converges on identity reconstruction as a core mechanism of recovery. Maruna (2001)’s landmark longitudinal research on desistance from crime and addiction demonstrated that individuals who successfully maintained abstinence had developed what he termed “redemption scripts,” coherent personal narratives that reframed past struggles as foundations for a transformed, agentic self. Best et al. (2016) formalized this insight in the Social Identity Model of Recovery (SIMOR), arguing that a critical driver of recovery is social identity transition: the psychological and social shift from “user” to “person in recovery.” This model helps explain why interventions such as Alcoholics Anonymous and employment programs prove effective. Their therapeutic power lies not primarily in behavioral techniques but in providing supportive social networks and positive role identities that enable individuals to author a new sense of self (Frings & Albery, 2015). Chen (2024) reinforced this perspective, demonstrating that identity construction and stigma management are pivotal factors in cessation trajectories. Recovery, in this view, is an extended process of biographical reconstruction wherein a stigmatized “substance user” identity is progressively replaced by a “non-user” identity anchored in valued social roles. Paris and Bradley (2001), in their research on alcohol recovery, similarly emphasized that identity renewal constitutes the central task of early recovery, a process intertwined with the restoration of authentic, reciprocal relationships. Collectively, these findings suggest that interventions targeting symptomatic relief without addressing the underlying identity crisis may achieve only partial and temporary gains.

Yet a significant gap persists between mechanistic understanding and intervention design. Although current treatment modalities demonstrate moderate effectiveness, they typically focus on symptom reduction rather than systematic identity transformation (Witkiewitz & Tucker, 2020). Much existing research remains confined to controlled laboratory or clinical settings, failing to capture how recovery unfolds within complex real-world social environments where stigma operates continuously. Structural barriers compound the problem: resource scarcity in treatment systems and pervasive societal stigma discourage help-seeking and undermine recovery maintenance, deepening the isolation that renders identity transformation difficult (Farhoudian et al., 2022; Kazdin, 2017; Volkow, 2020; Volkow & Collins, 2017). In sum, a pronounced “mechanism-intervention mismatch” characterizes the field. Identity reconstruction is increasingly recognized as important for recovery, yet interventions explicitly designed to engage this mechanism remain scarce.

Narrative Therapy (NT), developed by White and Epston (1990), offers a theoretically coherent response to this gap. The framework’s central premise is the separation of the person from the problem through a technique known as “externalizing.” Rather than treating addiction as an intrinsic trait (“I am an addict”), NT positions it as an external influence that has affected the person’s life (“Addiction has shaped my story”). This linguistic and cognitive reframing creates psychological distance, enabling individuals to examine their relationship with substances without the totalizing shame of identity fusion. Building on this foundation, NT employs “re-authoring” techniques to help individuals identify “unique outcomes” or “sparkling moments,” instances in their personal history that contradict the dominant problem-saturated narrative. These exceptions become the raw material for constructing an alternative life story, one emphasizing agency, resilience, and valued identities beyond addiction. This approach holds particular promise in Chinese cultural contexts, where concerns about “losing face” (mianzi) can intensify resistance to therapeutic disclosure (Mak et al., 2015). Externalizing techniques help bypass this cultural defense mechanism by allowing individuals to discuss addiction as something that happened to them rather than something they are. The problem becomes a third party in the conversation, preserving dignity while enabling therapeutic work.

Emerging empirical evidence supports the applicability of narrative-oriented interventions across stigmatized populations. Murengera et al. (2025) suggested NT is an effective framework for addressing psychological distress in genocide survivors through structured narrative reconstruction and community healing. Sun et al. (2022) found that NT decreased illness-related stigma and enhanced hope among oral cancer patients. In psychiatric rehabilitation, Yanos et al. (2011) showed that Narrative Enhancement and Cognitive Therapy (NECT), a group intervention incorporating re-authoring elements, reduced self-stigma and psychological distress among individuals with severe mental illness. Oudejans et al. (2022) provided further evidence supporting this conclusion, reporting that NECT effectively reduced self-stigma among individuals with severe mental illness in a Dutch pilot study. McAdams and McLean (2013) have argued, from a developmental psychology perspective, that constructing coherent life narratives integrating key experiences is the primary pathway through which individuals form a unified sense of identity.

Despite this promising evidence base, controlled evaluations of NT specifically targeting identity reconstruction in addiction populations remain limited. The theoretical alignment is compelling: NT’s emphasis on externalizing the problem and re-authoring life stories directly addresses the identity fusion and narrative impoverishment characteristic of chronic substance users. The present study was designed as an exploratory proof-of-concept pilot. Within an evidence-based practice (EBP) framework, it sought to obtain preliminary evidence on the feasibility, acceptability, and potential effects of an NT group intervention on early-stage identity consolidation and psychological relapse-risk indicators among individuals in residential addiction treatment. The aim was to generate initial data to inform the planning of larger, adequately powered confirmatory trials rather than to provide definitive evidence of efficacy. By examining both outcome and candidate process measures, we also sought to identify whether gains in self-concept clarity may warrant investigation as a process variable in future research.

## 2. Materials and Methods

### 2.1. Participants

Forty-five male participants were recruited from a city-run compulsory isolation drug rehabilitation center in Guangdong Province, China. Participants reside in a closed, custodial environment with restricted interaction with the outside world, ensuring no access to illicit substances during the study period. While admission to the facility is mandatory for residents, participation in this specific psychological intervention was strictly voluntary and independent of their release timing or legal standing. The mean age was 31.71 years (*SD* = 7.09). Recruitment occurred in May 2025 through collaboration between the research team and the facility administration.

Inclusion criteria were: (1) educational attainment at or above junior secondary level; (2) voluntary consent to participate in group psychological intervention; (3) history of two or more episodes of substance use, with primary substances being etomidate or metomidate derivatives; and (4) absence of literacy or cognitive impairments that would preclude participation in group activities and self-report measures. All participants demonstrated sufficient literacy to complete the written narrative components (e.g., letters, journals) during the screening phase.

### 2.2. Measures

Self-Identity Scale (SIS). Self-identity was assessed using the Self-Identity Scale developed by Ochse and Plug (1986). Grounded in Erikson’s psychosocial development theory, the instrument measures the degree to which individuals have established a coherent sense of identity. The scale comprises 19 items (e.g., “I know how I should live my life”; “I do not know what kind of person I am”) rated on a 4-point Likert scale ranging from 1 (never) to 4 (always). After reverse-scoring appropriate items, total scores are computed, with higher scores indicating stronger identity consolidation and lower scores suggesting role confusion or identity crisis. Internal consistency in the present sample was adequate (Cronbach’s α = 0.814).

Self-Concept Clarity Scale (SCCS). Self-concept clarity, serving as a process measure, was assessed using the Self-Concept Clarity Scale originally developed by Campbell et al. (1996). This unidimensional instrument evaluates the extent to which self-beliefs are clearly defined, internally consistent, and temporally stable. The 12 items are rated on a 5-point Likert scale from 1 (strongly disagree) to 5 (strongly agree), with higher scores reflecting greater clarity and coherence in self-perception. The scale demonstrated good internal consistency (Cronbach’s α = 0.844).

Relapse Risk Scale (RRS). Relapse propensity was assessed using an abbreviated Chinese version of the Stimulant Relapse Risk Scale (Geng et al., 2010; Ogai et al., 2007). The original instrument contains 20 items. To reduce participant burden and optimize administration efficiency for repeated measurement, a content-based core-item selection strategy was adopted, consistent with guidance on brief-scale construction (Boateng et al., 2018; Polit & Beck, 2006). The following four items were retained, each representing a key relapse-risk dimension: cue reactivity, negative affect, impulse dyscontrol, and subjective craving. A recent systematic review indicates that brief, psychometrically supported self-report tools have practical value in substance use research (Stewart et al., 2023). Sample items include “If someone offered me drugs, I would find it hard to refuse” and “I have no motivation to do anything.” Items are rated on a 5-point Likert scale (1 = strongly disagree, 5 = strongly agree), with higher scores indicating elevated psychological relapse risk. Internal consistency in the present sample was acceptable (Cronbach’s α = 0.774), supporting its use for between-group comparisons and longitudinal trend analysis in this pilot context (Tavakol & Dennick, 2011). We acknowledge that formal validation of this 4-item version has not been established, and results should be interpreted with appropriate caution.

### 2.3. Research Design

This pilot stratified randomized controlled trial employed a pre-post design with two-month follow-up assessment. The investigation was conducted between April and July 2025. The study employed an age-based stratified randomization method. Participants were first stratified into a “youth group” (≤30 years) and “middle-aged group” (>30 years) to control for developmental differences in identity formation and recovery trajectories. Within these strata, participants were randomly assigned to the Narrative Therapy (NT), Treatment-As-Usual (TAU), or waitlist control (WLC) conditions using a block randomization sequence to ensure balanced arm sizes (*n* = 15 per group). Randomization was performed using a block size of 3 within each stratum to ensure equal allocation (1:1:1) to the three arms.

All group facilitators possessed more than ten years of professional experience in group psychotherapy. Baseline assessments, including demographic information and all outcome and process measures, were completed prior to intervention commencement. The intervention phase consisted of eight weekly sessions, each lasting 90 min, conducted on Wednesday afternoons. Post-intervention assessments were administered immediately following the final session. No participant attrition occurred during the intervention phase; all groups maintained their original composition of 15 members. Follow-up assessments were conducted two months after intervention completion, with full retention across all conditions.

### 2.4. Intervention Protocols

#### 2.4.1. Evidence-Based Practice Framework

The intervention design adhered to an evidence-based practice (EBP) framework integrating the following three core components: best available research evidence, clinical expertise, and participant values and preferences (American Psychological Association Presidential Task Force on Evidence-Based Practice, 2006). The selection of Narrative Therapy was informed by a systematic review of the empirical literature documenting its efficacy in identity reconstruction and stigma reduction across clinical populations.

A comprehensive search strategy was implemented across English-language databases (Web of Science, PubMed, PsycINFO) and Chinese-language databases (CNKI, Wanfang Data) from inception through May 2025. Search terms combined intervention descriptors (“Narrative therapy” OR “Narrative exposure therapy” OR “Storytelling intervention” OR “Group psychotherapy”) with outcome and population descriptors (“Self-identity” OR “Ego-identity” OR “Stigma” OR “Internalized stigma” OR “Substance use” OR “Addiction”). This search yielded multiple randomized controlled trials and quasi-experimental studies, including Murengera et al. (2025) and Sun et al. (2022), which collectively informed the intervention protocol.

The theoretical framework drew primarily on McAdams and McLean (2013)’s narrative identity theory and White and Epston (1990)’s therapeutic techniques. Consistent with Maruna (2001)’s concept of “redemption scripts” in addiction recovery, identity reconstruction was established as the intervention’s primary therapeutic target.

#### 2.4.2. Narrative Therapy Group Intervention

The NT intervention protocol comprised eight structured sessions integrating core narrative therapeutic techniques. Table 1 presents the session-by-session framework.

#### 2.4.3. Treatment-as-Usual Group

The TAU condition followed the facility’s standard psychoeducational curriculum, designed to provide foundational psychological support and mental health literacy. Unlike the NT condition, this protocol was not grounded in any specific therapeutic orientation and excluded specialized techniques such as externalizing or re-authoring. This design controlled for placebo and Hawthorne effects.

The intervention format emphasized didactic instruction supplemented by general-purpose group activities. Facilitators delivered mental health content and provided basic guidance, focusing on cognitive education rather than deep psychological restructuring. The eight sessions addressed the following topics: (1) group formation and contracting; (2) self-awareness and exploration; (3) interpersonal communication skills; (4) emotion identification and regulation; (5) self-confidence enhancement; (6) intimate relationship patterns; (7) life and career planning; (8) review and termination.

### 2.5. Statistical Analysis

All analyses were performed using SPSS version 26.0. Normality was assessed via Shapiro–Wilk tests, and homogeneity of variance was examined using Levene’s test. Baseline equivalence was evaluated through one-way ANOVA. To test the group × time interaction directly, a 3 (group: NT, TAU, and WLC) × 3 (time: pre-test, post-test, and two-month follow-up) mixed-design repeated-measure ANOVA was conducted for each primary outcome. Simple-effect analyses followed significant interactions, with within-group effects across time and between-group effects at each time point examined separately. Effect sizes are reported as partial eta-squared (*η*^2^*p*). Given the small pilot sample, results are presented as preliminary estimates rather than confirmatory tests.

## 3. Results

### 3.1. Baseline Comparisons

Preliminary analyses confirmed that all variables met assumptions of normality and homogeneity of variance. One-way ANOVA revealed no significant baseline differences among the three groups on self-identity, *F*(2, 42) = 0.816, *p* = 0.449, or relapse risk, *F*(2, 42) = 0.798, *p* = 0.457 (Table 2). These findings indicated no statistically significant baseline differences among groups, supporting baseline comparability.

### 3.2. Repeated-Measure Analysis of Primary Outcomes

A 3 × 3 mixed-design repeated-measure ANOVA was conducted for each primary outcome (Table 3). For self-identity, the main effect of time was significant, *F* = 93.222, *p* < 0.001, *η*^2^*p* = 0.689, the main effect of group was significant, *F* = 19.266, *p* < 0.001, *η*^2^*p* = 0.478, and the group × time interaction was significant, *F* = 64.215, *p* < 0.001, *η*^2^*p* = 0.754. For relapse-risk indicators, the main effect of time was significant, *F* = 85.891, *p* < 0.001, *η*^2^*p* = 0.672, the main effect of group was significant, *F* = 11.823, *p* < 0.001, *η*^2^*p* = 0.360, and the group × time interaction was significant, *F* = 62.131, *p* < 0.001, *η*^2^*p* = 0.747. These patterns indicate that both outcomes changed differentially across conditions over time.

### 3.3. Simple-Effect Analyses

**Figure 1:**
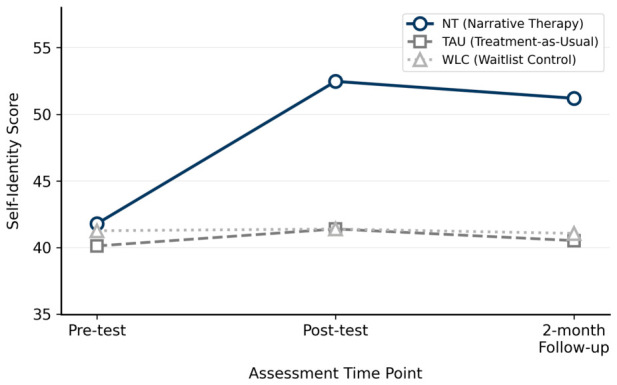
Changes in self-identity scores across the three groups at pre-test, post-test, and two-month follow-up.

Self-identity (Table 4). Baseline scores did not differ across groups, *F* = 0.816, *p* > 0.05, *η*^2^*p* = 0.037. At post-test and at two-month follow-up, between-group differences were significant, *F* = 61.686, *p* < 0.001, *η*^2^*p* = 0.746, and *F* = 39.852, *p* < 0.001, *η*^2^*p* = 0.655, respectively. Within-group analyses showed significant change only in the NT group, *F* = 131.042, *p* < 0.001, *η*^2^*p* = 0.903. Neither the TAU group, *F* = 0.651, *p* = 0.433, nor the WLC group, *F* = 3.500, *p* = 0.082, showed significant within-group change. Figure 1 displays the group trajectories in self-identity scores across the three time points.

**Figure 2:**
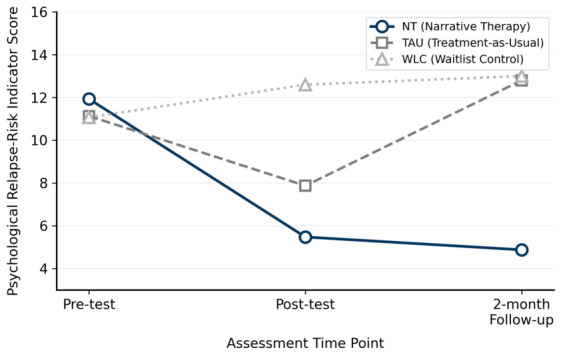
Changes in relapse-risk indicator scores across the three groups at pre-test, post-test, and two-month follow-up.

Relapse-risk indicators (Table 5). Baseline scores did not differ across groups, *F* = 0.798, *p* > 0.05, *η*^2^*p* = 0.037. Significant between-group differences emerged at post-test, *F* = 45.840, *p* < 0.001, *η*^2^*p* = 0.686, and at follow-up, *F* = 61.038, *p* < 0.001, *η*^2^*p* = 0.744. Within-group changes were significant in all three conditions, with the NT group showing the largest effect, *F* = 184.197, *p* < 0.001, *η*^2^*p* = 0.929, followed by the TAU group, *F* = 12.324, *p* = 0.003, *η*^2^*p* = 0.468, and the WLC group, *F* = 11.387, *p* = 0.005, *η*^2^*p* = 0.449. The TAU group showed a reduction at post-test followed by a return toward baseline at follow-up, while the WLC group’s scores rose across all three time points. The NT group’s large post-intervention reductions were sustained at the two-month follow-up, with no rebound toward baseline. Figure 2 displays the group trajectories in relapse-risk indicator scores across the three time points.

Pairwise post hoc comparisons were conducted using Tukey HSD as the primary test. Bonferroni-corrected comparisons served as a robustness check. Full pairwise results appear in Appendix A, Table A1. For self-identity, NT scores were significantly higher than both TAU and WLC at post-test (both *p* < 0.001). The same pattern was held at two-month follow-up (both *p* < 0.001). TAU and WLC did not differ at post-test (*p* = 1.000) or at follow-up (*p* = 0.917). For relapse-risk indicators, NT scores were significantly lower than both TAU (*p* = 0.008) and WLC (*p* < 0.001) at post-test. At follow-up, NT remained significantly lower than both TAU and WLC (both *p* < 0.001). TAU also scored significantly lower than WLC at post-test (*p* < 0.001). This short-term advantage was no longer present at follow-up (*p* = 0.969). The pattern suggests that the TAU group’s early reduction may have dissipated over the follow-up interval. Levene’s test indicated heterogeneity of variance for relapse-risk at post-test (*p* = 0.005) and at follow-up (*p* = 0.001). The Bonferroni-corrected results converged with Tukey across all comparisons, supporting robustness.

### 3.4. Process Evaluation: Self-Concept Clarity

Paired-sample t-tests assessed changes in self-concept clarity within the NT and TAU groups (Table 6). The NT group exhibited significant pre-post improvement on the Self-Concept Clarity Scale, *t*(14) = −2.368, *p* < 0.05. The TAU group showed no significant change, *t*(14) = −1.131, *p* > 0.05. The between-group difference in change scores did not reach significance, *F*(2, 42) = 2.494, *p* = 0.095.

## 4. Discussion

The present findings provide preliminary, exploratory evidence that Narrative Therapy group intervention may produce improvements in self-identity among individuals in residential addiction treatment, with parallel reductions in self-reported psychological relapse-risk indicators. The repeated-measure analysis yielded a significant group × time interaction for both outcomes, and simple-effect tests showed that only the NT group exhibited significant within-group gains. These effects were not observed in the TAU or WLC conditions and were maintained at two-month follow-up. The pattern extends beyond general symptom relief toward what accumulating evidence identifies as a core psychological structure underlying recovery—the reconstruction of a coherent, non-stigmatized identity. The results offer initial, tentative support for the Social Identity Model of Recovery (Best et al., 2016; Frings & Albery, 2015). Because the study was conducted in a closed custodial setting where participants had no access to substances, the relapse-risk findings reflect short-term psychological vulnerability (cue reactivity, negative affect, impulse dyscontrol, and subjective craving) rather than actual relapse behavior. Results should therefore be read as evidence of change in psychological risk indicators under controlled conditions, not as confirmation of relapse-prevention efficacy.

### 4.1. Identity Reconstruction as a Cognitive Coping Scaffold

Understanding how the intervention may have shaped these effects requires examining identity reconstruction not merely as an outcome but as a cognitive coping scaffold that reorganizes how individuals process addiction-related challenges. Participants entered treatment with a fused self-concept in which personal identity had collapsed into the stigmatized category of “addict.” This fusion may have generated a self-perpetuating cycle as follows: every craving, every slip, every social rejection confirms the pathological self-narrative and erodes motivation for change. NT intervention may have disrupted this cycle at its foundation. Through externalizing conversations, participants may have engaged in cognitive defusion. They possibly loosened the automatic equivalence between self and substance use (White & Epston, 1990). The addiction may have been repositioned as an external influence rather than an intrinsic trait. This linguistic shift created psychological distance, enabling participants to evaluate their relationship with substances without paralyzing shame. Once this separation was established, the excavation of unique outcomes provided concrete biographical evidence contradicting the dominant problem-saturated narrative. These “sparkling moments,” instances of resistance and agency that had been rendered invisible by the totalizing addict identity, became cognitive anchoring points for an alternative self-story.

The process measure findings offer preliminary insight into a hypothesized pathway linking these cognitive shifts to identity outcomes. While within-group improvements in Self-Concept Clarity were observed for the NT group, the between-group difference in change scores was not statistically significant. Therefore, Self-Concept Clarity should be viewed as a candidate process variable rather than a confirmed mediator. The within-group pattern is theoretically suggestive: clarity may precede stability. Before individuals can consolidate a new identity, they must first develop a coherent internal representation of who they are and who they wish to become. The NT intervention, by guiding participants to integrate past experiences, present values, and future aspirations into a unified narrative, may have established what McAdams (2001) terms “biographical continuity.” This temporal integration addresses a core vulnerability in addiction: the tendency to escape an intolerable present by returning to substance use. When past suffering is reframed as the foundation for current strength and future possibility, the psychological pressure toward relapse may diminish.

### 4.2. The Group as Transformative Social Context

While Section 4.1 addressed internal cognitive shifts, the group format may have provided supportive external conditions for these changes to take hold. Consistent with classical formulations of group dynamics (Yalom, 2005), the homogeneous composition may have fostered a destigmatized social environment that could have helped reduce chronic isolation. Each act of self-disclosure, each expression of mutual support, likely generated immediate positive feedback. Through repeated cycles of such interaction, external social connection may have become internalized as psychological resilience (Witkiewitz & Tucker, 2020). The group may have functioned as an intermediary space, a rehearsal context where participants could experiment with new relational patterns before transferring them to broader social environments.

Two specific techniques deserve elaboration for their potential role in mobilizing group dynamics toward identity transformation.

**Re-Membering Conversations.** The Week 5 intervention was designed to mobilize relational resources to support identity change. Participants identified significant others and reconstructed these relationships as sources of validation rather than judgment. This technique populates what White (2007) describes as the “club of life” with supportive internalized audiences. Even when physically isolated, individuals carrying such internalized relationships possess external reference points for sustaining their emergent self-concept.

**Definitional Ceremony.** The culminating Week 7 and Week 8 sessions were intended to provide social legitimation for reconstructed identities. Facility staff and family members witnessed participants’ public declaration of their new selves. Recognition from valued others may have conferred social legitimacy on what had been private cognitive work, potentially transforming subjective aspiration into social fact. This ceremonial structure addresses a fundamental challenge: internal psychological shifts remain vulnerable to erosion unless anchored in external social reality.

### 4.3. Limitations and Future Directions

Several limitations warrant acknowledgment and should inform interpretation of these preliminary findings.

First, the total sample was relatively small (*N* = 45), and no a priori power analysis was conducted. Statistical power could be limited, particularly for the candidate process variable (self-concept clarity), where the between-group comparison did not reach significance (*p* = 0.095). The large effect-size estimates for self-identity and relapse-risk indicators should therefore be read as exploratory, not confirmatory, and may be inflated relative to true population effects.

Second, facilitators were not blinded to the study hypothesis. Different facilitators delivered the NT and TAU conditions. Observed effects may partly reflect facilitator enthusiasm, differential therapeutic alliance, or participant expectancy rather than the narrative techniques alone. Future trials should adopt facilitator randomization, crossed or counterbalanced facilitator designs, and process measures of therapeutic alliance and treatment expectancy, to better separate specific technique effects from common factors.

Third, while we observed significant within-group improvements in Self-Concept Clarity for the NT condition, we could not statistically confirm this candidate process variable as the causal mechanism of change. Formal mediation analyses with larger samples are needed to determine whether self-concept clarity functions as a process mechanism or as a correlate of change.

Fourth, the abbreviated four-item relapse-risk measure, although internally consistent in this sample, has not undergone formal validation. Replication with the full scale or a psychometrically validated short form is recommended.

Fifth, the sample was drawn exclusively from male participants at a single residential facility, constraining ecological validity. Whether comparable effects would emerge in community-based settings, among female populations, or with different substance use profiles remains to be established. These constraints represent opportunities for future research on gender-specific narrative interventions and the differential effectiveness of NT across treatment contexts.

Sixth, and importantly, the closed custodial context meant that outcomes reflected perceived vulnerability rather than observed substance use. The results may indicate short-term psychological protection, but they cannot speak to real-world relapse prevention. Longer prospective designs tracking participants through the high-risk post-discharge phase, with behavioral outcomes, are needed before conclusions about relapse prevention can be drawn.

## 5. Conclusions

This exploratory pilot stratified randomized controlled trial provides preliminary evidence that a Narrative Therapy group intervention may promote early-stage identity consolidation among individuals in residential addiction treatment. A significant group × time interaction was observed for both outcomes, and pairwise post hoc tests confirmed that NT scores were significantly more favorable than both control groups at post-test and follow-up. NT reductions in relapse-risk indicators were maintained at two-month follow-up, whereas TAU’s short-term advantage over WLC had dissipated by follow-up. Within-group gains in self-concept clarity suggest a candidate process pathway, although between-group differences on this variable did not reach significance.

These findings are best viewed as preliminary and hypothesis-generating rather than confirmatory. The small sample, single-site design, unblinded delivery, use of an unvalidated abbreviated scale, and custodial assessment context may limit the strength and generalizability of the conclusions, particularly regarding actual relapse prevention. Future research would benefit from larger, multi-site randomized controlled trials, with adequate power, active control conditions matched for therapeutic engagement, post-discharge behavioral outcomes, and formal mediation analyses. Should future adequately powered studies replicate these preliminary patterns, narrative-oriented interventions may hold promise as one component of broader efforts to support more holistic approaches to addiction care.

## Figures and Tables

**Table 1:** Narrative Therapy group intervention protocol.

Week	Theme	Therapeutic Objectives	Activities
1—Building Connection	Opening the Narrative Journey	Establish group trust; construct life timeline	“Story behind your name” sharing exercise to honor personal uniqueness; life timeline mapping (peak and valley experiences); collaborative development of group covenant
2—Who Am I?	Externalizing Conversations	Separate person from problem; deconstruct pathologizing labels	Metaphor exercise: “What is addiction like? How did it enter your life?”; label-tearing ritual: writing and destroying the “addict” label; deconstructive questioning
3—Finding Exceptions	Identifying Unique Outcomes	Excavate positive exceptions within recovery journey	Journal sharing: “Three moments in the past month when I overcame craving”; collaborative “I am a hero” story-building based on exceptional experiences
4—Re-Authoring My Life	Re-Storying	Reconstruct self-narrative	Cross-temporal dialog: letter to future self; role-play: enacting re-authored story segments
5—Re-Membering and Values	Re-Membering Conversations	Engage significant others as witnesses; clarify core values; mobilize social support	Identify five most important people; explore how they perceive the self; examine one’s influence on their lives; “Tree of Life” drawing to visualize values and relational connections
6—Meaning-Making	Integration and Reconciliation	Transform painful experiences into growth resources	Autobiographical writing: composing a letter of understanding to one’s past self, acknowledging persistence and change, achieving reconciliation; small-group sharing in triads
7—Identity Construction	Definitional Ceremony	Consolidate identity transition; establish long-term support network	“New Identity Launch”: public declaration of transformed self, with facility staff and willing family members invited as witnesses; presentation of “New Identity Certificate” with commemorative photograph
8—Closure and Continuation	From Author to Narrator	Review therapeutic journey; transform personal growth into transmissible resources	Exhibition of artifacts created during intervention (life timelines, future self-portraits); participant narration of key turning points (small-group discussion followed by selected public sharing); presentation of “Narrative Practitioner Certificate,” authorizing recipients to share their experiences with other individuals in recovery

**Table 2:** Baseline comparison across groups (*M* ± *SD*).

Variable	NT Group	95% CI	TAU Group	95% CI	WLC Group	95% CI	*F*	*p*
Self-Identity	41.80 ± 3.51	[40.0, 43.6]	40.13 ± 3.38	[38.4, 41.8]	41.27 ± 4.03	[39.2, 43.3]	0.816	0.449
Relapse Risk	11.93 ± 2.28	[10.8, 13.1]	11.13 ± 1.96	[10.1, 12.1]	11.07 ± 2.02	[10.1, 12.1]	0.798	0.457

Note. NT = Narrative Therapy; TAU = Treatment-As-Usual; WLC = waitlist control. CI = confidence interval.

**Table 3:** Repeated-measure ANOVA for self-identity and relapse-risk indicators.

Variable	Time *F* (*p*, *η*^2^*p*)	Group *F* (*p*, *η*^2^*p*)	Time × Group *F* (*p*, *η*^2^*p*)
Self-Identity	93.222 (<0.001, 0.689)	19.266 (<0.001, 0.478)	64.215 (<0.001, 0.754)
Relapse Risk	85.891 (<0.001, 0.672)	11.823 (<0.001, 0.360)	62.131 (<0.001, 0.747)

**Table 4:** Simple-effect analysis for self-identity (*M* ± *SD*).

Group	Pre (*M* ± *SD*)	Pre 95% CI	Post 1 (*M* ± *SD*)	Post 1 95% CI	Post 2 FU (*M* ± *SD*)	Post 2 FU 95% CI	Within-Group F (*p*, *η*^2^*p*)
WLC	41.27 ± 4.03	[39.04, 43.50]	41.40 ± 3.38	[39.53, 43.27]	41.07 ± 3.79	[38.97, 43.16]	3.500 (0.082, 0.200)
TAU	40.13 ± 3.38	[38.26, 42.00]	41.40 ± 2.64	[39.94, 42.86]	40.53 ± 3.85	[38.40, 42.67]	0.651 (0.433, 0.044)
NT	41.80 ± 3.51	[39.86, 43.74]	52.47 ± 3.38	[50.60, 54.34]	51.20 ± 3.41	[49.31, 53.09]	131.042 (<0.001, 0.903)

Note. NT = Narrative Therapy; TAU = Treatment-As-Usual; WLC = waitlist control. CI = confidence interval.

**Table 5:** Simple-effect analysis for relapse-risk indicators (*M* ± *SD*).

Group	Pre (*M* ± *SD*)	Pre 95% CI	Post 1 (*M* ± *SD*)	Post 1 95% CI	Post 2 FU (*M* ± *SD*)	Post 2 FU 95% CI	Within-Group F (*p*, *η*^2^*p*)
WLC	11.07 ± 2.02	[9.95, 12.18]	12.60 ± 2.97	[10.95, 14.25]	13.00 ± 2.83	[11.43, 14.57]	11.387 (0.005, 0.449)
TAU	11.13 ± 1.96	[10.05, 12.22]	7.87 ± 1.64	[6.96, 8.78]	12.80 ± 2.70	[11.30, 14.30]	12.324 (0.003, 0.468)
NT	11.93 ± 2.28	[10.67, 13.20]	5.47 ± 1.19	[4.81, 6.12]	4.87 ± 0.74	[4.46, 5.28]	184.197 (<0.001, 0.929)

Note. NT = Narrative Therapy; TAU = Treatment-As-Usual; WLC = waitlist control. CI = confidence interval.

**Table 6:** Pre-post self-concept clarity comparisons (*M* ± *SD*).

Variable	Timepoint	NT Group	95% CI	TAU Group	95% CI
Self-Concept Clarity	Pre	35.87 ± 3.52	[34.1, 37.7]	36.33 ± 2.77	[34.9, 37.7]
	Post	38.73 ± 2.22	[37.6, 39.9]	37.73 ± 4.65	[35.4, 40.1]
	t	−2.368 *		−1.131	

Note. * *p* < 0.05. CI = confidence interval.

## Data Availability

Data Security and Confidentiality Agreement: Based on the confidentiality agreement signed with the partners, access to certain data is strictly restricted.

## Appendix A

**Table A1:** Pairwise post hoc comparisons (Tukey HSD and Bonferroni) for self-identity and relapse-risk indicators at post-test and two-month follow-up.

Outcome	Time Point	Comparison (I vs. J)	Mean Diff (I − J)	Tukey 95% CI	Tukey *p*	Bonferroni *p*
Self-Identity	Post-test	NT vs. TAU	+11.07	[8.27, 13.86]	<0.001	<0.001
Self-Identity	Post-test	NT vs. WLC	+11.07	[8.27, 13.86]	<0.001	<0.001
Self-Identity	Post-test	TAU vs. WLC	0.00	[−2.80, 2.80]	1.000	1.000
Self-Identity	Follow-up	NT vs. TAU	+10.67	[7.40, 13.94]	<0.001	<0.001
Self-Identity	Follow-up	NT vs. WLC	+10.13	[6.86, 13.40]	<0.001	<0.001
Self-Identity	Follow-up	TAU vs. WLC	−0.53	[−3.80, 2.74]	0.917	1.000
Relapse-Risk	Post-test	NT vs. TAU	−2.40	[−4.24, −0.56]	0.008	0.009
Relapse-Risk	Post-test	NT vs. WLC	−7.13	[−8.98, −5.29]	<0.001	<0.001
Relapse-Risk	Post-test	TAU vs. WLC	−4.73	[−6.58, −2.89]	<0.001	<0.001
Relapse-Risk	Follow-up	NT vs. TAU	−7.93	[−9.97, −5.89]	<0.001	<0.001
Relapse-Risk	Follow-up	NT vs. WLC	−8.13	[−10.17, −6.09]	<0.001	<0.001
Relapse-Risk	Follow-up	TAU vs. WLC	−0.20	[−2.24, 1.84]	0.969	1.000

Note. NT = Narrative Therapy; TAU = Treatment-As-Usual; WLC = waitlist control. CI = confidence interval.
